# Pathologic studies of fatal cases in outbreak of hand, foot, and mouth disease, Taiwan.

**DOI:** 10.3201/eid0701.700146

**Published:** 2001

**Authors:** W J Shieh, S M Jung, C Hsueh, T T Kuo, A Mounts, U Parashar, C F Yang, J Guarner, T G Ksiazek, J Dawson, C Goldsmith, G J Chang, S M Oberste, M A Pallansch, L J Anderson, S R Zaki

**Affiliations:** Centers for Disease Control and Prevention, 1600 Clifton Road, Mail Stop G32, Atlanta, GA 30333, USA. wshieh@cdc.gov

## Abstract

In 1998, an outbreak of enterovirus 71-associated hand, foot, and mouth disease occurred in Taiwan. Pathologic studies of two fatal cases with similar clinical features revealed two different causative agents, emphasizing the need for postmortem examinations and modern pathologic techniques in an outbreak investigation.


### Pathologic Studies of Fatal Cases in Outbreak of Hand, Foot, and Mouth Disease, Taiwan

**Wun-Ju Shieh,* Shih-Ming Jung,† Chuen Hsueh,† Tseng-Tong Kuo,† Anthony Mounts,* Umesh Parashar,* Chen Fu Yang,* Jeannette Guarner,* Thomas G. Ksiazek,* Jacqueline Dawson,* Cynthia Goldsmith,* Gwong-Jen J. Chang,* Steve M. Oberste,* Mark A. Pallansch,* Larry J. Anderson,* Sherif R. Zaki,* and the Epidemic Working Group**[1]

*Centers for Disease Control and Prevention, Atlanta, Georgia, USA; and †Chang Gung Memorial Hospital, Tauyuan, Taiwan, Republic of China

In 1998, an outbreak of enterovirus 71-associated hand, foot, and mouth disease occurred in Taiwan. Pathologic studies of two fatal cases with similar clinical features revealed two different causative agents, emphasizing the need for postmortem examinations and modern pathologic techniques in an outbreak investigation.

During April through July 1998, an outbreak of hand, foot, and mouth disease occurred in Taiwan; enterovirus 71 (EV71) was identified as the main etiologic agent. The outbreak was associated with an unusually high death rate in young children. At least 55 fatal cases were initially reported (1,2) in healthy children, who had refractory shock after an acute prodromal illness; many of them manifested neurologic disorders during illness and died within 24 hours of hospitalization (3). In many fatal cases, EV71 was indicated as the etiologic agent by serologic, virologic, and polymerase chain reaction tests conducted on specimens from nonsterile sites, such as throat swabs or stool specimens. While these results may indicate a precedent EV71 infection in the fatal cases, they do not directly implicate EV71 as the causative agent of neurologic disorders and eventual death. In contrast, histopathologic examination in conjunction with special pathology techniques, such as immunohistochemistry (IHC), in situ hybridization, and electron microscopy, can provide unequivocal evidence linking a particular agent to death. We report results of pathologic examination of two fatal cases during this outbreak.

Address for correspondence: Wun-Ju Shieh, Centers for Disease Control and Prevention, 1600 Clifton Road, Mail Stop G32, Atlanta, GA 30333, USA: fax: 404-639-3043; e-mail: wshieh@cdc.gov.

### The Study

The clinical and histopathologic features of the first case were initially reported by Chang et al. (4); the second case was in a patient admitted to the same hospital with a similar clinical course. Histopathologic features were similar in both cases and showed severe and extensive encephalomyelitis. An IHC technique using a monoclonal mouse anti-EV71 antibody and an in situ hybridization test using a digoxigenin-labeled enterovirus probe were performed on formalin–fixed, paraffin-embedded central nervous system (CNS) tissues and major organs of both patients. In the first case, positive staining of enteroviral antigens and nucleic acids was observed in neurons, neuronal processes, and inflammatory foci at various CNS sites, including the cerebral cortex, brain stem, and all levels of spinal cord (Figure 1A, B). No immunostaining or hybridization was present in lung, heart, liver, spleen, or kidney. Electron microscopy evaluation of spinal cord tissues showed a highly vacuolated neuron containing scattered picornavirus-like particles and viral inclusions (Figure 1C).

[1]Epidemic Working Group at Centers for Disease Control and Prevention: Jim Alexander, Betty Brown, David Shay, Patricia Greer, Charles Humphrey, Tim Morken, and Tara Ferebee-Harris.

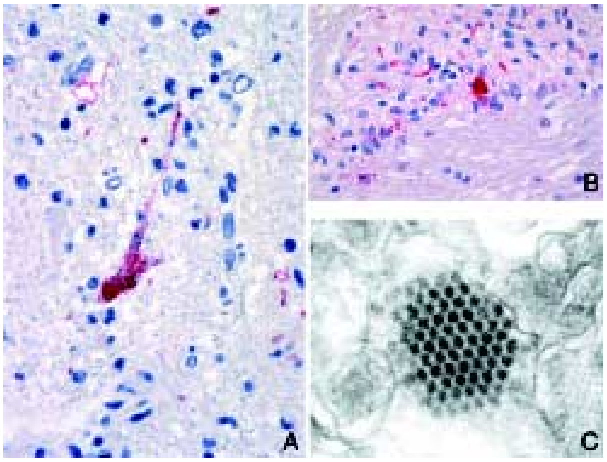

Figure 1. A). Positive immunostaining of EV71 antigens in neuron and neuronal process. Original magnification, X158. B). Positive immunostaining of EV71 antigens in necrotic area. Original magnification, X158. C). An array of picornavirus particles in a neuron (electron micrograph).

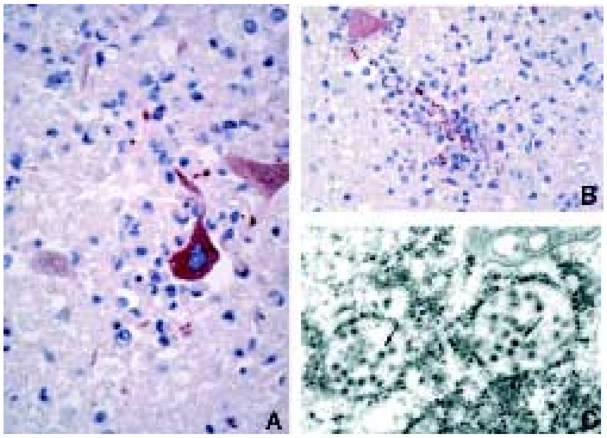

Figure 2. A). Positive immunostaining of Japanese encephalitis antigens in neuron and neuronal process. Original magnification, X158. B). Positive immunostaining of Japanese encephalitis antigens in necrotic area. Original magnification, X158. C). Flavivirus particles (arrowheads) in isolates from mouse brain (electron micrograph).

The second case was negative for EV71 by IHC, in situ hybridization, polymerase chain reaction, and viral isolation. An IHC test using an anti-Japanese encephalitis antibody showed intense immunostaining of flaviviral antigens in neurons, neuronal processes, and inflammatory foci at various CNS sites (Figure 2A, B). No immunostaining of flavivirus was present in other major organs. Further study was conducted by injecting neurologic tissue of the patient into suckling mice. CNS material from an inoculated mouse was passed onto Vero-E6 cells and produced cytopathic effect. Electron microscopy examination of mouse brain and infected cell culture revealed flavivirus particles (Figure 2C), and polymerase chain reaction with sequencing confirmed the isolate as Japanese encephalitis virus.

### Conclusions

In addition to classic hand, foot, and mouth disease, EV71 can cause severe CNS infections with a high death rate (5). Two previous EV71 outbreaks were associated with neurologic disorders and increased deaths; the first one occurred in Bulgaria in 1975 (5) and the second in Malaysia in 1997 (1,6). The neurologic disorders and clinical courses of acute viral CNS infections can be very similar regardless of causative agents, and definitive diagnoses are sometimes very difficult to establish without further pathologic studies. A definitive laboratory and pathologic diagnosis is crucial for implementing effective measures to control an outbreak (7); the Nipah virus encephalitis outbreak in Malaysia (8,9) and the West Nile encephalitis outbreak in New York City (10,11) lend additional support to this statement.

Our report illustrates how two different etiologic agents can cause similar diseases during an outbreak and emphasizes the need for postmortem examination.

Dr. Shieh is a staff pathologist, Division of Viral and Rickettsial Diseases, Centers for Disease Control and Prevention. His research interests include infectious disease pathology, pathology and pathogenesis of viral encephalitides, molecular epidemiology, and infectious disease outbreak investigations.

### References

1. Centers for Disease Control and Prevention. Deaths among children during an outbreak of hand, foot, and mouth disease –Taiwan, Republic of China, April-July 1998. MMWR Morb Mortal Wkly Rep 1998;47:629-32.
2. Ho M, Chen ER, Hsu KH, Twu SJ, Chen KT, Tsai SF, et al. An epidemic of enterovirus 71 infection in Taiwan. Taiwan Enterovirus Epidemic Working Group. N Engl J Med 1999;341:929-35.
3. Liu C, Tseng H, Wang S, Wang J, Su I. An outbreak of enterovirus 71 infection in Taiwan, 1998: epidemiologic and clinical manifestations. J Clin Virol 2000;17:23-30.
4. Chang LY, Huang YC, Lin TL. Fulminant neurogenic pulmonary oedema with hand, foot, and mouth disease. Lancet 1998;352: 367-8.

5. Shindarov L, Chumakov M, Voroshilova M, Bojinov S, Vasilenko SM, Iordanov I, et al. Epidemiological, clinical and pathomorphological characteristics of epidemic poliomyelitis-like disease caused by enterovirus 71. J Hyg Epidemiol Microbiol Immunol 1979; 23: 284-95.
6. Lum LCS, Wong KT, Lam SK, Chua KB, Goh AY, Lim WL, et al. Fatal enterovirus 71 encephalomyelitis. J Pediatr 1998;133:795-8.
7. Zaki SR, Shieh W-J, and the Epidemic Working Group at Ministry of Health in Nicaragua, et al. Leptospirosis associated with outbreak of acute febrile illness and pulmonary haemorrhage, Nicaragua, 1995. Lancet 1996;347:535-6.
8. Centers for Disease Control and Prevention. Outbreak of Hendra-like virus—Malaysia and Singapore, 1998–1999. MMWR Morb Mortal Wkly Rep 1999;48:265–9.
9. Chua KB, Bellini WJ, Rota PA, Harcourt BH, Tamin A, Lam SK, et al. Nipah virus: a recently emergent deadly Paramyxovirus. Science 2000;288:1432-5.
10. Centers for Disease Control and Prevention. Outbreak of West Nile-like viral encephalitis--New York, 1999. MMWR Morb Mortal Wkly Rep 1999;48:845-9.
11. Shieh WJ, Guarner J, Layton M, Fine A, Miller J, Nash D, et al. The role of pathology in an investigation of an outbreak of West Nile encephalitis in New York, 1999. Emerg Infect Dis 2000;6:370-2.
